# Potential of Predatory Bacteria to Colonize the Duckweed Microbiome and Change Its Structure: A Model Study Using the Obligate Predatory Bacterium, *Bacteriovorax* sp. HI3

**DOI:** 10.1264/jsme2.ME23040

**Published:** 2023-09-08

**Authors:** Daisuke Inoue, So Nakamura, Tomomi Sugiyama, Michihiko Ike

**Affiliations:** 1 Division of Sustainable Energy and Environmental Engineering, Osaka University, 2–1 Yamadaoka, Suita, Osaka 565–0871, Japan

**Keywords:** duckweed, microbiome alteration, predatory bacteria, *Bacteriovorax*

## Abstract

Modifying the duckweed microbiome is a major challenge for enhancing the effectiveness of duckweed-based wastewater treatment and biomass production technologies. We herein examined the potential of the exogenous introduction of predatory bacteria to change the duckweed microbiome. *Bacteriovorax* sp. HI3, a model predatory bacterium, colonized the core of the *Lemna* microbiome, and its predatory behavior changed the microbiome structure, which correlated with colonization density. These results reveal that bacterial predatory interactions may be important drivers that shape the duckweed microbiome, suggesting their potential usefulness in modifying the microbiome.

Duckweed (Lemnaceae family) is a small floating aquatic plant that is widely distributed in nutrient-rich freshwater environments (Cao *et al.*, 2020). Due to its beneficial properties, such as very high growth capability, tolerance to various pollutants and other abiotic stresses, and valuable biomass content, wastewater treatment systems using duckweed have emerged as economical and sustainable alternatives for the treatment of wastewater and generation of value-added products (Singh *et al.*, 2023). Recent studies demonstrated that duckweed is colonized by a microbiome, which is composed of diverse bacteria, with the dominance of several core taxa (Acosta *et al.*, 2020; Ishizawa *et al.*, 2020; Iwashita *et al.*, 2020; Inoue *et al.*, 2022a). In addition, individual bacteria in the microbiome may exert positive or negative effects on the growth and physiological status of the host plant (Ishizawa *et al.*, 2017), which affects the wastewater treatment and biomass production capabilities of duckweed-based wastewater treatment systems. Therefore, microbiome control is a possible means of enhancing their effectiveness, but remains a major challenge due to complicated interactions within the microbiome and between the microbiome and host plant as well as dynamic changes under contrasting environmental conditions.

We herein propose the use of predatory bacteria as a strategy to control the duckweed microbiome. Predatory bacteria are a group of bacteria that actively lyse other bacteria and consume their cell-derived macromolecules as nutrients (Pérez *et al.*, 2016). In natural environments, predatory bacteria play a significant role in shaping microbial communities, carbon and nutrient cycling, and energy flow through microbial food webs (Hungate *et al.*, 2021; Petters *et al.*, 2021; Mookherjee and Jurkevitch, 2022). In addition, their lytic activities are only effective against a specific range of bacteria and harmless to eukaryotic organisms, including plants and animals (Waso *et al.*, 2021; Mookherjee and Jurkevitch, 2022). Therefore, there has been a growing interest in their biotechnological application, including the biocontrol of specific pathogens (Bratanis *et al.*, 2020; Waso *et al.*, 2021).

To date, few studies have used predatory bacteria to regulate microbial communities. In addition, to the best of our knowledge, the colonization and fate of predatory bacteria and the effects of bacterial predator-prey interactions on aquatic plants, including duckweed, remain unclear. We herein focused on *Bdellovibrio* and like organisms (BALOs), which are obligate predatory bacteria, and investigated (1) whether an exogenously introduced BALO strain may successfully establish itself in the duckweed (*Lemna* spp.) microbiome, and (2) how and to what extent the introduction of this strain affects the microbiome and subsequent growth of duckweed.

To construct the duckweed microbiome, surface water samples from three freshwater ponds (A, B, and C) in Osaka, Japan (Table S1) were used as microbial sources. After filtration through a 3.0-μm filter (Merck Millipore) to remove coarse particles, including fungi and microalgae, microbes in the samples were recovered by centrifugation (10,000×*g*, 4°C, 10‍ ‍min), washed twice with modified Hoagland (MH) medium (Toyama *et al.*, 2006), and resuspended in MH medium to obtain equivalent concentrations to the original pond water. Ten fronds of a laboratory stock of sterilized duckweed (*Lemna* RDSC clone 5512), which was cultured with MH medium in a growth chamber (28°C, photon flux of 80‍ ‍μmol m^–2^ s^–1^, 16‍ ‍h light cycle), were transferred to 60‍ ‍mL of the prepared microbial suspension in a 100-mL flask and cultured for 8‍ ‍d under the same conditions as described above to allow the duckweed microbiome to stabilize (Acosta *et al.*, 2020; Ishizawa *et al.*, 2020). *Lemna* assembled with the microbiome from ponds A, B, and C were hereafter designated as LMb-A, LMb-B, and LMb-C, respectively.

*Bacteriovorax* sp. HI3, which was previously isolated from pond water (Inoue *et al.*, 2022b), was used as the model BALO strain. Strain HI3 was routinely cultivated at 28°C on a HM buffer-based double-layer agar plate, the top layer of which was inoculated with *Escherichia coli* HB101 as prey (Inoue *et al.*, 2022b). HM buffer consisted of 25‍ ‍mM HEPES, 3‍ ‍mM CaCl_2_, and 2‍ ‍mM MgCl_2_ (pH 7.4). When HI3 was used for experiments, a segment of the double-layer agar plate with a lytic halo formed by HI3 was homogenized using a disposable homogenizer (Biomasher II; Nippi), inoculated into a preculture of *E. coli* HB101 in R2A broth (DAIGO; Nihon Pharmaceutical), and cultured at 28°C for 72‍ ‍h with rotary shaking at 120‍ ‍rpm. The culture was then filtered twice through a 0.45-μm membrane filter (Advantec) to remove prey cells. Since the cells of BALOs and *Escherichia* spp. are generally 0.25 to 0.5×0.75 to 1.25‍ ‍μm (Jurkevitch, 2012) and 1.1 to 1.5×2.0 to 6.0‍ ‍μm (Scheutz and Strockbine, 2015) in size, respectively, some HI3 cells pass through a 0.45-μm filter, whereas prey cells are completely trapped. HI3 cells remaining in the filtrate were harvested by centrifugation (10,000×*g*, 4°C, 10‍ ‍min), washed twice with MH medium, and resuspended in 60‍ ‍mL of MH medium in a 100-mL flask at 5.0×10^6^‍ ‍cells‍ ‍mL^–1^. Ten fronds of LMb-A, LMb-B, or LMb-C were transferred to the flasks. After a 5-d cultivation period (*i.e.*, the 1st batch cultivation), ten *Lemna* fronds were transplanted into fresh MH medium. This procedure was repeated twice to complete three-batch cultivation. Control experiments without the HI3 inoculation, as well as with transplanting axenic *Lemna*, were also performed. All treatments were conducted in triplicate. The overall procedure of the cultivation experiments is shown in Fig. S1.

To monitor transitions in the abundance of HI3 and structure of the bacterial community in the *Lemna* microbiome during sequential batch cultivations, *ca.* eight *Lemna* fronds were collected at the end of the 1st and 3rd batch cultivations and preserved at –80°C. The duckweed sample was immersed in 100‍ ‍μL of Cica Geneus DNA extraction solution (Kanto Chemical), and microbial DNA was isolated according to the manufacturer’s protocol. The abundance of strain HI3 was quantified using qPCR targeting the partial 16S rRNA gene sequence, as previously reported (Inoue *et al.*, 2022b). Amplification efficiency was 93.5–100.8% and the correlation coefficient was 0.996–0.999. A dissociation curve ana­lysis found a specific peak at 84.5°C in all of the samples analyzed, which confirmed the negligible level of non-HI3 sequences that may be amplified by the qPCR primers used in the present study. The DNA copies obtained using qPCR were converted to cell numbers based on two copies of the 16S rRNA gene being present in the HI3 genome (AP026946).

The entire bacterial community was analyzed by 16S rRNA amplicon sequencing targeting the V5–V6 region (Inoue *et al.*, 2022a). Sequencing was performed on an Illumina MiSeq platform. Sequence reads were processed using QIIME 2 (ver. 2022.2). The Greengenes database (ver. 13.8) was used for the taxonomic assignment of amplicon sequence variants (ASVs). The taxonomic assignment of specific ASVs was also conducted using an NCBI BLAST search (https://blast.ncbi.nlm.nih.gov/Blast.cgi). The α-diversity of the bacterial communities was evaluated using the Shannon index. To further examine the effects of HI3 on‍ ‍the *Lemna* microbiome, co-occurrence networks were constructed by grouping all of the ASV datasets into two‍ ‍groups, *i.e.*, with and without the inoculation of HI3.‍ ‍Each group consisted of six samples (three pond water samples×two sampling periods). Only ASVs with >100 reads in all 12 samples were included in the matrix calculation using the Matrix package in R (ver. 4.0.5). A relationship was considered to be significant when Spearman |ρ|>0.75 and *P*<0.05. Networks were further analyzed and visualized using Gephi ver. 0.9.7 (Bastian *et al.*, 2009).

During sequential batch cultivation, the inoculation with HI3 generally did not affect the growth of *Lemna* positively or negatively from that without the HI3 inoculation (Fig. S2). Although the growth of axenic *Lemna* appeared to be suppressed in the 3rd batch after the inoculation with HI3, this was not a direct effect of the inoculation with HI3 because the density of H13 was below detection levels by the 3rd batch (Fig. 1A). These results are consistent with previous findings showing that BALOs were harmless to eukaryotic organisms (Waso *et al.*, 2021; Mookherjee and Jurkevitch, 2022).

Fig. 1A shows the colonization density of HI3 on *Lemna*. HI3 inoculated into axenic *Lemna* declined to 2.1×10^3^ cells (g wet weight)^–1^ in the 1st batch, reaching below the detection limit (<2.4×10^2^ cells [g wet weight]^–1^) in the 3rd batch. In contrast, HI3 persisted on *Lemna* that had been colonized with pond water-derived microbiomes during the three batch cultivations. This indicated the colonization ability of the obligate predator HI3 on *Lemna* by actively preying on its precolonized bacteria. Predation by BALOs may efficiently occur on biofilms, which are one form of microorganisms existing on the duckweed surface (Yamaga *et al.*, 2010; Ishizawa *et al.*, 2019; Tan *et al.*, 2021); therefore, biofilms are considered a common niche for BALOs (Kadouri and O’Toole, 2005; Mookherjee and Jurkevitch, 2022), which is consistent with the present results.

However, the fate of HI3 in the three *Lemna* microbiomes differed from each other: from the 1st to 3rd batches, it increased from 1.2×10^5^ to 3.3×10^5^ cells (g wet weight)^–1^ in LMb-A, declined from 2.0×10^4^ to 1.3×10^3^ cells (g wet weight)^–1^ in LMb-B, and remained at 2.4×10^4^ cells (g wet weight)^–1^ in LMb-C (Fig. 1A). In 16S rRNA sequencing, ASV_006, which was not detected in the original *Lemna* microbiome and had a 100% identical 16S rRNA gene sequence to HI3 (AP026946), was also detected at a higher relative abundance in LMb-A than in LMb-B and LMb-C (Fig. 1B). In contrast, 16S rRNA sequencing detected no BALO ASVs in LMb-A and one *Bacteriovorax* ASV in LMb-B and LMb-C. ASV_079 and ASV_029, which were detected in LMb-B and LMb-C, respectively, were the most closely related to *Bacteriovorax stolpii* DSM 12778 (NR_042023; identity, 98% [381 bp/387 bp] and 99% [383‍ ‍bp/387 bp], respectively), which was also the closest relative of ASV_006. In LMb-B, another BALO ASV (ASV_023), which was closely related to *Bacteriovorax* sp. EPA (AY294220; identity, 95% [369 bp/387 bp]) and *Peredibacter starrii* A3.12 (NR_024943; identity, 95% [367‍ ‍bp/387 bp]), was also detected. Among these ASVs, the relative abundance of ASV_023 in LMb-B was not affected by the HI3 inoculation. In contrast, ASV_079 in LMb-B and ASV_029 in LMb-C disappeared in the 3rd batch after the inoculation with HI3; however, their abundance remained relatively stable in the controls without the HI3 inoculation. These results suggest the following hypotheses on the colonization of exogenous BALOs in the *Lemna* microbiome. If indigenous BALOs are absent from the *Lemna* microbiome, it likely lacks resistance and is, thus, highly susceptible to bacterial predation. Consequently, in this *Lemna* microbiome, exogenously introduced BALOs may be readily capable of developing an ecological niche through vigorous predation, as observed in LMb-A in the present study. Furthermore, when BALOs originally present in the *Lemna* microbiome are taxonomically similar and likely have similar prey ranges to those exogenously introduced, exogenous BALOs may partially replace them, as observed in LMb-B and LMb-C. In this case, the colonization level of exogenous BALOs may be restricted owing to competition with indigenous BALOs for the same prey and the inherent predation resistance of the indigenous bacteria (Shemesh and Jurkevitch, 2004). Furthermore, the decline in the abundance of HI3 in LMb-B may have been affected by the high abundance of non-prey bacteria that potentially act as decoys (Sathyamoorthy *et al.*, 2021), in addition to inherent predation resistance.

Fig. 1B shows the composition of the *Lemna* microbiome with and without the HI3 inoculation as a heat map. The‍ ‍overall structures of the *Lemna* microbiome are also‍ ‍provided as a bar chart in Fig. S3. Previous studies demonstrated that *Caulobacteraceae*, *Comamonadaceae*, *Flavobacteriaceae*, *Methylophilaceae*, *Oxalobacteraceae*, and *Sphingomonadaceae* were commonly and dominantly present in duckweed microbiomes (Acosta *et al.*, 2020; Ishizawa *et al.*, 2020; Inoue *et al.*, 2022a). These families were also dominant in the three *Lemna* microbiomes before the inoculation with HI3, accounting for 70.2–88.4% of the‍ ‍total communities. Their dominance was maintained throughout the cultivation experiment, irrespective of the HI3 inoculation (68.9–92.5% and 61.0–90.0% with and without the HI3 inoculation, respectively). The diversity of the *Lemna* microbiome after the HI3 inoculation was similar overall to that of the control microbiome (Table S2). Exceptionally, the diversity of LMb-C after the HI3 inoculation markedly declined in the 3rd batch, which was attributed to the high collective dominance of the six aforementioned families (*i.e.*, 92.5%) and the lower ASV number than that in the corresponding control (Table S2). The specific reasons for the dominance of particular taxa after the introduction of HI3 in the specific *Lemna* microbiome (*i.e.*, LMb-C) remain unclear. Further studies are needed to resolve this issue and obtain a more detailed understanding of the microbiome-modulating/altering effects of BALOs.

In addition, a marked increase or decrease in the abundance of several specific taxa was observed depending on the colonization of HI3 (Fig. 1B). *Pseudomonadaceae* commonly showed a larger decline in the HI3-inoculated microbiome than that in the control microbiome (*e.g.*, in the 1st batch, 0 and 32.3% in LMb-A, 2.3 and 7.6% in LMb-B, and 0 and 3.0% in LMb-C with and without the HI3 inoculation, respectively). Furthermore, in LMb-C, *Oxalobacteraceae* were diminished by the inoculation with HI3. In contrast, the inoculation with HI3 in LMb-A resulted in a larger abundance of *Rhodocyclaceae* than the control without the HI3 inoculation. Furthermore, the change in the abundance of *Comamonadaceae* after the HI3 inoculation differed among the three *Lemna* microbiomes: smaller, similar, and larger abundance than the corresponding controls in LMb-A, LMb-B, and LMb-C, respectively. Consequently, variations in these specific taxa led to a shift in the overall structure of the *Lemna* microbiome through the colonization of HI3, with the most prominent changes in the LMb-A microbiome representing the largest HI3 colonization (Fig. 1B and S3). To further examine the interactions between HI3 and other members of the *Lemna* microbiome, co-occurrence networks were constructed from the ASV dataset with and without the HI3 inoculation. The results obtained revealed that ASV_006, identified as HI3, was included in the community with an average degree of 26, which was higher than that of the other communities (1.0–20) (Fig. 2B and 2D). Collectively, these results indicated that exogenously introduced HI3 had intensive connections with the core members of the *Lemna* microbiome and contributed to the regulation of its structure. Indigenous BALO ASVs in the duckweed microbiome (*i.e.*, ASV_023 and ASV_079) were also allocated to the community with the highest average degree in the control microbiome (Fig. 2A and 2C), emphasizing the importance of predatory bacteria in the *Lemna* microbiome.

Based on the results of the co-occurrence network ana­lysis, ASVs showing a correlation with ASV_006 were identified (Table S3). ASV_007, ASV_100, and ASV_167 negatively correlated with ASV_006. These ASVs appeared to be susceptible to direct predation by HI3, as suggested by Cohen *et al.* (2021). Despite the common decline in *Pseudomonadaceae* following the inoculation with HI3 (Fig. 1B), no ASV assigned to the family showed a negative correlation with ASV_006 (Table S3). This discrepancy was caused by the presence of different *Pseudomonadaceae* ASVs in the three *Lemna* microbiomes. However, these results collectively suggest that members of *Pseudomonadaceae* were commonly susceptible to HI3. In contrast, 15 ASVs positively correlated with ASV_006, and may be indirectly affected by the predation of other members of the microbiome by HI3. Their density may increase by utilizing the leftover cell components generated by bacteriolysis by HI3 or by the acquisition of an ecological niche in the *Lemna* microbiome corresponding to declines in their competitors due to predation by HI3. Similarly, the ASVs that negatively correlated with ASV_023 and ASV_079 were only five and one out of 29 and 11 ASVs, respectively (Table S4 and S5), all of which were different from the three ASVs that negatively correlated with ASV_006 (Table S3). In contrast, ASV_005 and ASV_040, which had negative correlations with ASV_023 and ASV_079, respectively, positively correlated with ASV_006. Three ASVs that positively correlated with ASV_079 (*i.e.*, ASV_077, ASV_119, and ASV_166) also had positive correlations with ASV_006, while this commonality was not observed between ASV_023 and ASV_006 (Table S3, S4, and S5), which may be attributed to taxonomic closeness and, thus, a similar prey range between ASV_006 and ASV_079. The few ASVs that had a negative correlation with ASV_006 and other ASVs representing indigenous BALOs may be attributable to the small number of sequencing samples and also to the assembly of the results from the three *Lemna* microbiomes. Therefore, further detailed studies with more samples from specific cultivation systems are required to identify populations that are directly susceptible to bacterial predation.

In conclusion, the present study revealed that obligate predatory bacteria colonize the core of the duckweed microbiome and also that interactions via bacterial predation may be important drivers shaping the microbiome structure, which corroborated the applicability of the exogenous introduction of predatory bacteria in modifying the duckweed microbiome. Unfortunately, duckweed growth was not markedly improved by the introduction of HI3 in the present study. However, since plant growth-promoting bacteria and plant growth-inhibiting bacteria (PGIB) coexist in the duckweed microbiome (Ishizawa *et al.*, 2017), one strategy to successfully promote duckweed growth by applying predatory bacteria may be the selective elimination of PGIB. Therefore, the identification of specific predatory bacteria capable of selectively preying on PGIB is an important issue that needs to be resolved. The present results also emphasize the importance of identifying interactions via bacterial predation to comprehensively elucidate the mechanisms underlying the assembly and transitions of the duckweed microbiome. Predatory bacteria will be key to developing both ecological studies on and the engineering of duckweed microbiomes.

All raw sequence reads related to the present study are available in the DDBJ Sequence Read Archive under the accession number DRA015989.

## Citation

Inoue, D., Nakamura, S., Sugiyama, T., and Ike, M. (2023) Potential of Predatory Bacteria to Colonize the Duckweed Microbiome and Change Its Structure: A Model Study Using the Obligate Predatory Bacterium, *Bacteriovorax* sp. HI3. *Microbes Environ ***38**: ME23040.

https://doi.org/10.1264/jsme2.ME23040

## Supplementary Material

Supplementary Material

## Figures and Tables

**Fig. 1.**
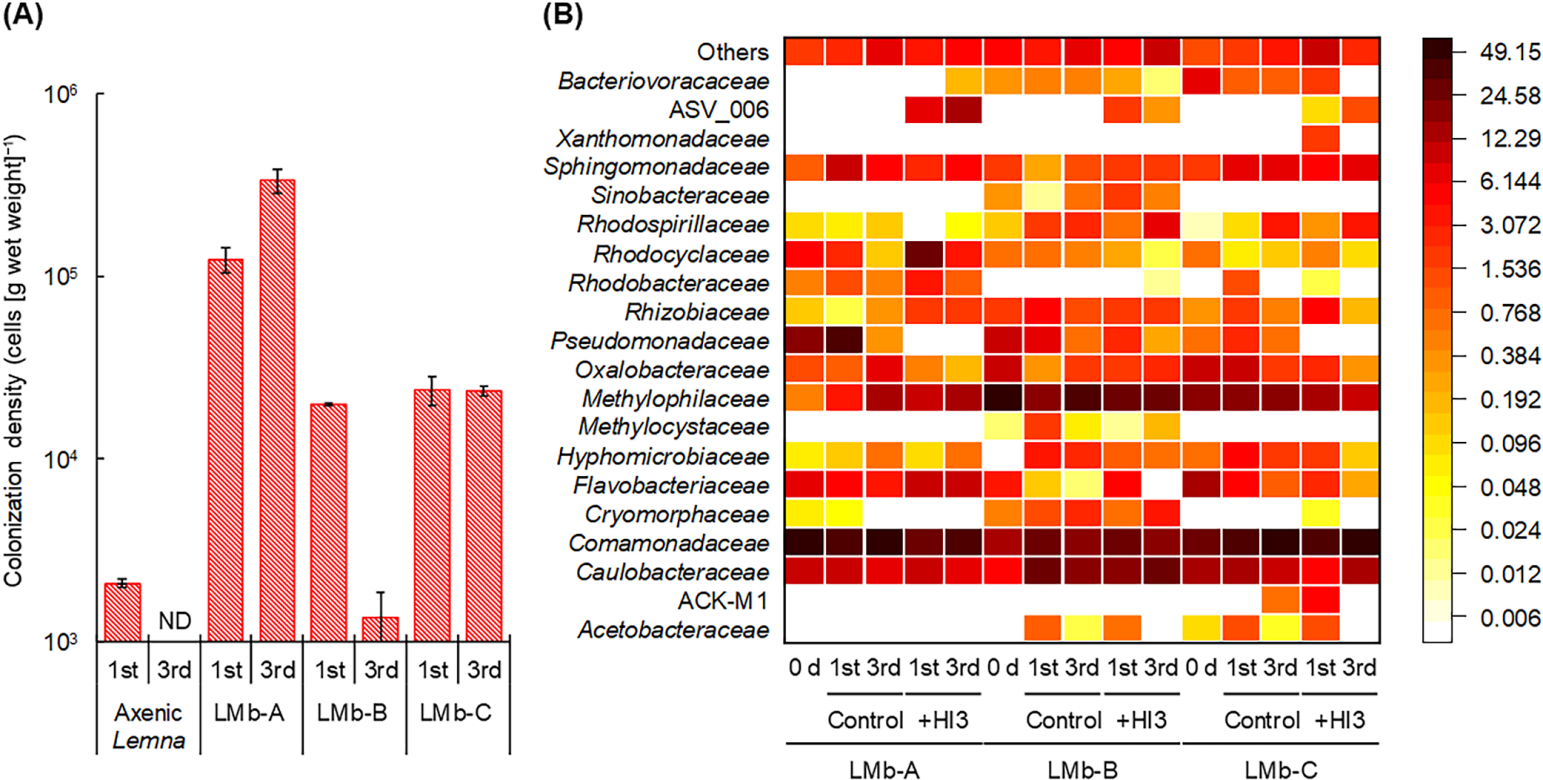
Temporal variations in the colonization of *Bacteriovorax* sp. HI3 and bacterial community compositions on *Lemna*. (A) Colonization density of *Bacteriovorax* sp. HI3 on axenic *Lemna* and on *Lemna* assembled with microbiomes from pond water A (LMb-A), B (LMb-B), and C (LMb-C). ND: <2.4×10^2^ cells (g wet weight)^–1^. (B) Heat map showing the relative abundance of bacterial families in the three *Lemna* microbiomes with and without the inoculation of *Bacteriovorax* sp. HI3. ASV_006 identified as *Bacteriovorax* sp. HI3 is shown independently. Unassigned families and families with relative abundance <1.0% are assembled as “Others”.

**Fig. 2.**
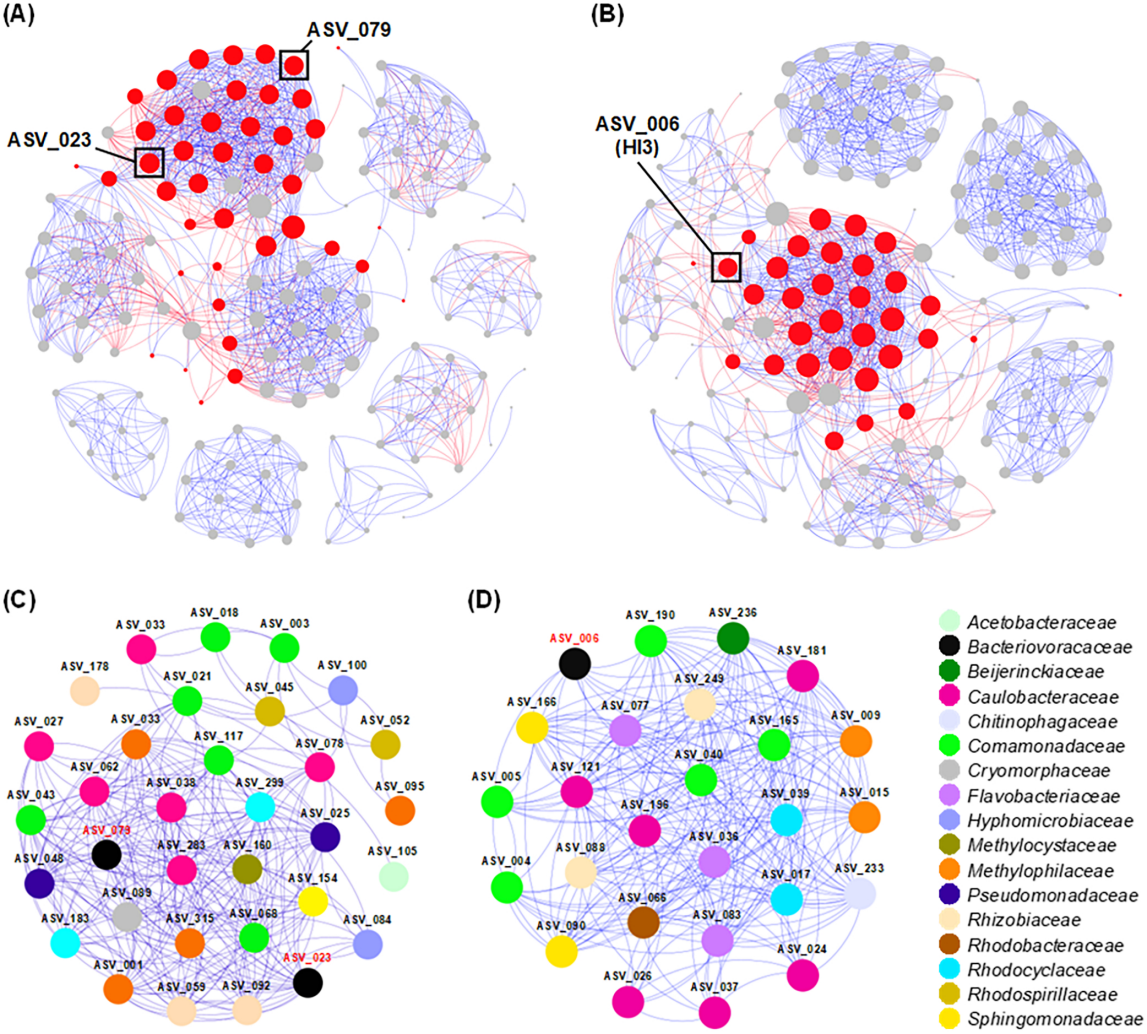
Co-occurrence network ana­lysis of the *Lemna* microbiome without (A, C) and with (B, D) the inoculation of *Bacteriovorax* sp. HI3. Blue- and red-colored edges indicate positive and negative correlations, respectively (Spearman |ρ|>0.75 and *P*<0.05). (A, B) The overall co-occurrence network. The node size is proportional to the degree of the node, and red-colored nodes indicate the communities with the highest average degree. (C, D) Correlations within the communities with the highest average degree. The node size is constant, irrespective of the degree of the node.
