# Supplementary material for: Potential of Predatory Bacteria to Colonize the Duckweed Microbiome and Change Its Structure: A Model Study Using the Obligate Predatory Bacterium, Bacteriovorax sp. HI3
Source: Microbes Environ. 2023 Sep 8;38(3):ME23040. doi: 10.1264/jsme2.ME23040 (PMC10522839; doi:10.1264/jsme2.ME23040)
Supplement: Supplementary file 1 — Supplementary Material [file 38_23040_s1.pdf]

Table S1. Pond water samples used as microbial sources

Pond	Location		Water temperature (°C)	pH
	Latitude	Longitude		
A	34°48'36"N	135°30'24"E	18.8	7.70
B	34°46'14"N	135°28'55"E	18.9	6.87
C	34°48'08"N	135°31'11"E	20.5	7.31

Table S2. Summary of 16S rRNA sequencing analysis of the duckweed microbiome

Pond	Sample	Read No.	ASV No.	Good's coverage	Shannon index
A	0 d	37,106	71	0.998	2.64
	Control – 1st batch	33,615	72	0.998	2.56
	Control – 3rd batch	37,925	90	0.998	3.12
	+ HI3 – 1st batch	34,257	73	0.998	2.83
	+ HI3 – 3rd batch	35,362	81	0.998	3.04
B	0 d	38,455	95	0.998	2.63
	Control – 1st batch	35,335	66	0.998	3.13
	Control – 3rd batch	39,631	100	0.997	2.93
	+ HI3 – 1st batch	36,927	95	0.997	3.32
	+ HI3 – 3rd batch	39,625	100	0.997	3.23
C	0 d	40,318	70	0.998	3.06
	Control – 1st batch	38,997	68	0.998	3.28
	Control – 3rd batch	38,264	94	0.998	3.10
	+ HI3 – 1st batch	39,303	83	0.998	3.34
	+ HI3 – 3rd batch	38,106	78	0.998	2.44

Table S3. ASVs showing significant positive or negative correlation with ASV_006 in the co-occurrence network analysis

ASV ID	Correlation	Closest relative	
		Family	Genus
ASV_134	+	*Boseaceae*	*Bosea*
ASV_121	+	*Caulobacteraceae*	*Phenylobacterium*
ASV_181	+	*Caulobacteraceae*	*Asticcacaulis*
ASV_004	+	*Comamonadaceae*	*Acidovorax*
ASV_005	+	*Comamonadaceae*	*Limnohabitans*
ASV_007	–	*Comamonadaceae*	*Diaphorobacter*
ASV_040	+	*Comamonadaceae*	*Hydrogenophaga*
ASV_155	+	*Comamonadaceae*	*Rhodoferax*
ASV_190	+	*Comamonadaceae*	*Acidovorax*
ASV_100	–	*Devosiaceae*	*Devosia*
ASV_167	–	*Fimbriimonadaceae*	*Fimbriimonas*
ASV_077	+	*Flavobacteriaceae*	*Flavobacterium*
ASV_236	+	*Pleomorphomonadaceae*	*Methylobrevis*
ASV_249	+	*Rhizobiaceae*	*Pararhizobium*
ASV_119	+	*Sphingobacteriaceae*	*Pedobacter*
ASV_090	+	*Sphingomonadaceae*	*Novosphingobium*
ASV_091	+	*Sphingomonadaceae*	*Sphingobacterium*
ASV_166	+	*Sphingomonadaceae*	*Sphingomonas*

Table S4. ASVs showing significant positive or negative correlation with ASV_023 in the co-occurrence network analysis

ASV ID	Correlation	Closest relative	
		Family	Genus
ASV_079	+	*Bacteriovoracaceae*	*Bacteriovorax*
ASV_024	–	*Caulobacteraceae*	*Brevundimonas*
ASV_026	–	*Caulobacteraceae*	*Brevundimonas*
ASV_038	+	*Caulobacteraceae*	*Brevundimonas*
ASV_062	+	*Caulobacteraceae*	*Asticcacaulis*
ASV_078	+	*Caulobacteraceae*	*Asticcacaulis*
ASV_283	+	*Caulobacteraceae*	*Asticcacaulis*
ASV_089	+	*Chitinophagaceae*	*Taibaiella*
ASV_005	–	*Comamonadaceae*	*Rhodoferax*
ASV_117	+	*Comamonadaceae*	*Aquabacterium*
ASV_084	+	*Devosiaceae*	*Devosia*
ASV_106	+	*Kaistiaceae*	*Prosthecomicrobium*
ASV_001	+	*Methylophilaceae*	*Methylophilus*
ASV_015	–	*Methylophilaceae*	*Pseudomethylobacillus*
ASV_109	+	*Methylophilaceae*	*Methylovorus*
ASV_315	+	*Methylophilaceae*	*Methylobacillus*
ASV_160	+	*Pleomorphomonadaceae*	*Oharaeibacter*
ASV_025	+	*Pseudomonadaceae*	*Pseudomonas*
ASV_042	+	*Pseudomonadaceae*	*Pseudomonas*
ASV_048	+	*Pseudomonadaceae*	*Pseudomonas*
ASV_059	+	*Rhizobiaceae*	*Rhizobium*
ASV_092	+	*Rhizobiaceae*	*Pararhizobium*
ASV_126	+	*Rhizobiaceae*	*Rhizobium*
ASV_150	+	*Rhizobiaceae*	*Peteryoungia*
ASV_043	+	*Sphaerotilaceae*	*Aquincola*
ASV_102	–	*Sphingomonadaceae*	*Sphingomonas*
ASV_154	+	*Sphingomonadaceae*	*Novosphingobium*
ASV_183	+	*Sterolibacteriaceae*	*Georgfuchsia*
ASV_299	+	*Zoogloeaceae*	*Uliginosibacterium*

Table S5. ASVs showing significant positive or negative correlation with ASV_079 in the co-occurrence network analysis

ASV ID	Correlation	Closest relative	
		Family	Genus
ASV_196	+	*Caulobacteraceae*	*Asticcacaulis*
ASV_040	–	*Comamonadaceae*	*Hydrogenophaga*
ASV_077	+	*Flavobacteriaceae*	*Flavobacterium*
ASV_083	+	*Flavobacteriaceae*	*Flavobacterium*
ASV_009	+	*Methylophilaceae*	*Methylotenera*
ASV_214	+	*Pseudomonadaceae*	*Pseudomonas*
ASV_143	+	*Rhizobiaceae*	*Shinella*
ASV_119	+	*Sphingobacteriaceae*	*Pedobacter*
ASV_166	+	*Sphingomonadaceae*	*Sphingomonas*
ASV_253	+	*Sphingomonadaceae*	*Chakrabartia*
ASV_320	+	*Sphingomonadaceae*	*Novosphingobium*

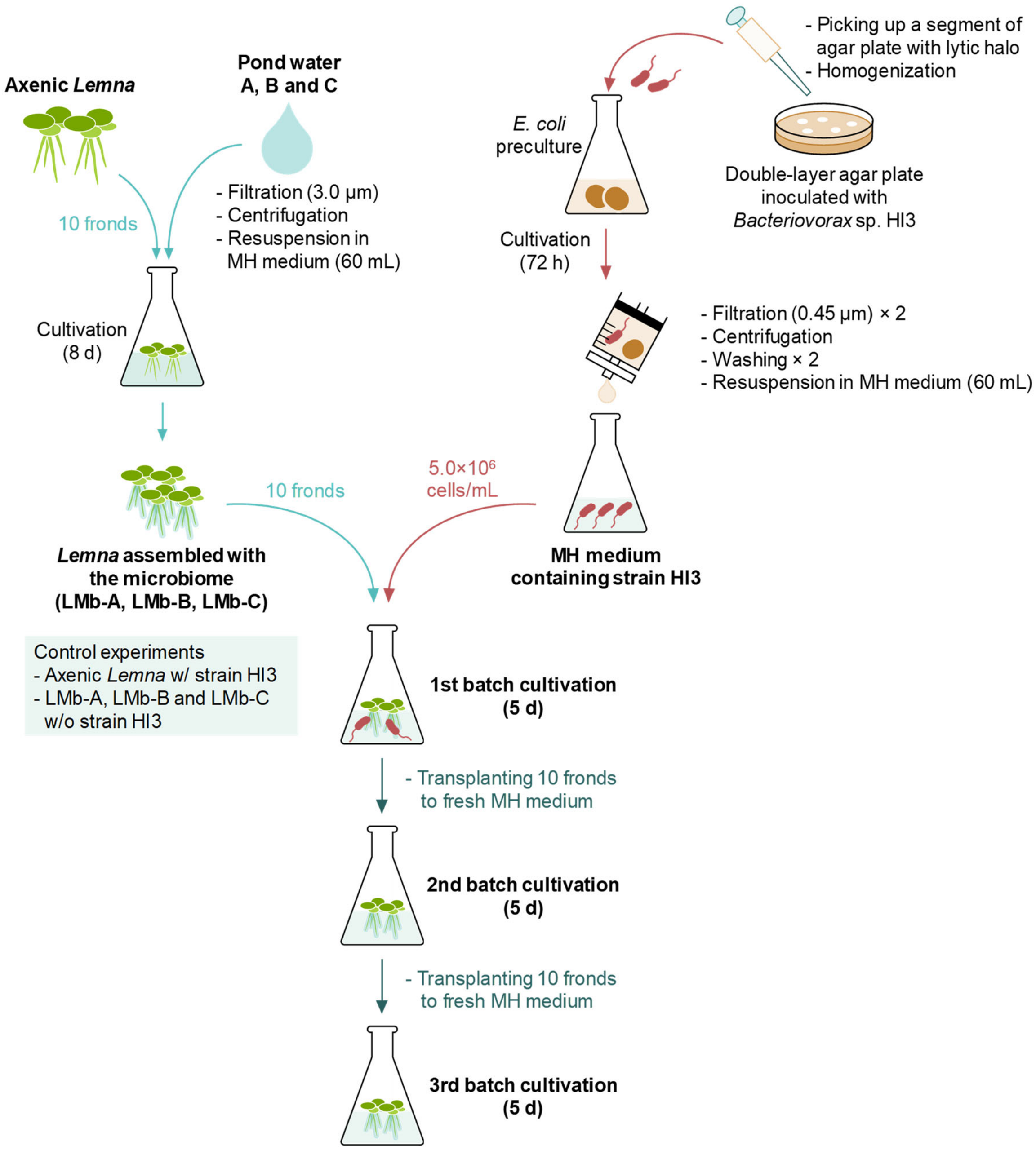

Fig. S1. Overall procedure of the cultivation experiments performed in this study, including the construction of the duckweed microbiome, preparation of *Bacteriovorax* sp. HI3, and co-cultivation of the duckweed microbiome and *Bacteriovorax* sp. HI3.

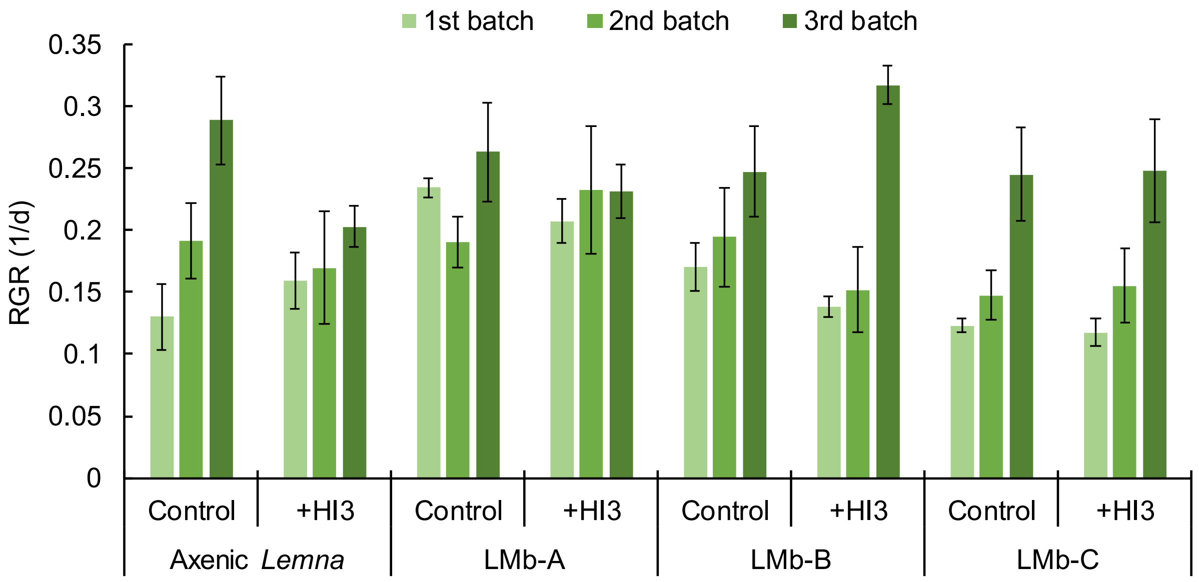

Fig. S2. Growth of *Lemna* with and without colonization by the pond water-derived microbiome and inoculation with *Bacteriovorax* sp. HI3. The relative growth rate (RGR) was used as an indicator of plant growth: RGR (1/d) = (ln N_2 – ln N_1)/T, where N_1 and N_2 are the number of fronds at the beginning and end of batch cultivation, respectively, and T is the length of batch cultivation. Error bars indicate standard deviation (n = 3).

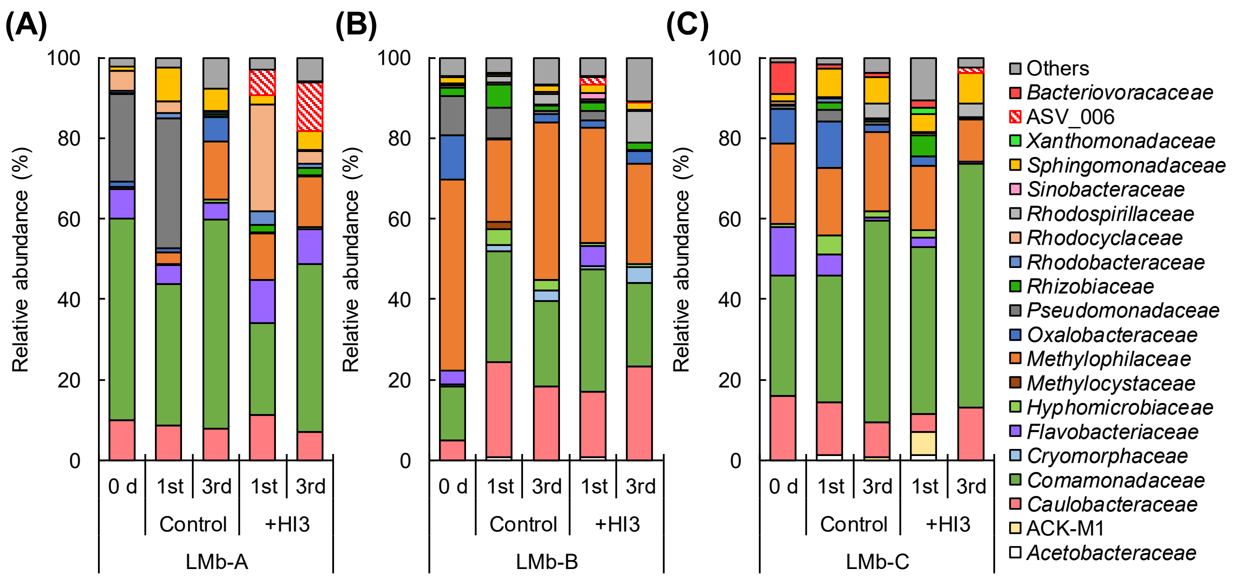

Fig. S3. Composition of the bacterial community at the family level in LMb-A (A), LMb-B (B), and LMb-C (C), with and without inoculation of *Bacteriovorax* sp. HI3. ASV_006 identified as *Bacteriovorax* sp. HI3 is shown independently. Unassigned families and families with relative abundance <1.0% are assembled as "Others".
